# Low-Dose Decitabine Assists Human Umbilical Cord-Derived Mesenchymal Stem Cells in Protecting *β* Cells via the Modulation of the Macrophage Phenotype in Type 2 Diabetic Mice

**DOI:** 10.1155/2020/4689798

**Published:** 2020-04-03

**Authors:** Jing Xue, Yu Cheng, Haojie Hao, Jieqing Gao, Yaqi Yin, Songyan Yu, Junyan Zou, Jiejie Liu, Qi Zhang, Yiming Mu

**Affiliations:** ^1^Medical School of Chinese PLA, Beijing, China; ^2^Department of Endocrinology, The First Medical Center of Chinese PLA General Hospital, Beijing, China; ^3^Department of Molecular Biology, Institute of Basic Medicine, School of Life Science, Chinese PLA General Hospital, Beijing, China

## Abstract

**Background:**

Progressive *β*-cell dysfunction, a major characteristic of type 2 diabetes (T2D), is closely related to the infiltration of inflammatory macrophages within islets. Mesenchymal stem cells (MSCs) have been identified to alleviate *β*-cell dysfunction by modulating macrophage phenotype in T2D, but the restoration of *β*-cells by a single MSC infusion is relatively transient. Decitabine (DAC) has been reported to polarize macrophages towards the anti-inflammatory phenotype at low doses. We therefore investigated whether low-dose decitabine could enhance the antidiabetic effect of MSCs and further promote the restoration of *β*-cell function.

**Methods:**

We induced a T2D mice model by high-fat diets and streptozotocin (STZ) injection. Mice were divided into five groups: the normal group, the T2D group, the DAC group, the MSC group, and the MSC plus DAC group (MD group). We examined the blood glucose and serum insulin levels of mice 1, 2, and 4 weeks after MSC and/or DAC treatment. Dynamic changes in islets and the phenotype of intraislet macrophages were detected via immunofluorescence. In vitro, we explored the effect of MSCs and DAC on macrophage polarization.

**Results:**

The blood glucose and serum insulin levels revealed that DAC prolonged the antidiabetic effect of MSCs to 4 weeks in T2D mice. Immunofluorescence staining demonstrated more sustainable morphological and structural amelioration in islets of the MD group than in the MSC group. Interestingly, further analysis showed more alternatively activated macrophages (M2, anti-inflammatory) and fewer classically activated macrophages (M1, proinflammatory) in islets of the MD group 4 weeks after treatment. An in vitro study demonstrated that DAC together with MSCs further polarized macrophages from the M1 to M2 phenotype via the PI3K/AKT pathway.

**Conclusion:**

These data unveiled that DAC prolonged the antidiabetic effect of MSCs and promoted sustainable *β*-cell restoration, possibly by modulating the macrophage phenotype. Our results offer a preferable therapeutic strategy for T2D.

## 1. Introduction

Progressive decline in *β*-cell mass and function, a major characteristic of type 2 diabetes (T2D), will inevitably lead to insulin deficiency as the disease progresses [1]. Existing treatments such as insulin secretagogues can temporarily increase insulin secretion, but similar to exogenous insulin supplements, they are unable to reverse progressive *β*-cell dysfunction [2]. In addition, despite the possible role of dipeptidyl peptidase-IV (DPP-IV) inhibitors and glucagon-like peptide-1 (GLP-1) receptor agonists in enhancing *β*-cell function in rodents, the effect of these drugs on human islets has not been fully confirmed [3, 4]. This highlights the need to find new therapies targeting the fundamental pathogenesis of T2D.

Mesenchymal stem cells (MSCs) are fibroblast-like adult stem cells. In addition to their self-renewable and multipotent differentiation ability, MSCs also possess broad immunoregulatory properties and anti-inflammatory effects [5–8]. Notably, small-sample clinical trials and experimental studies have demonstrated that MSC infusion could preserve functional *β*-cell mass and ameliorate hyperglycemia in T2D individuals [9–15]. Bhansali et al. reported that autologous MSC transplantation decreased insulin requirements and elevated stimulated C-peptide levels in T2D patients in a prospective, randomized, single-blinded placebo-controlled study [15]. Si et al. [9] found T2D rats exhibited improved glycemic homeostasis and islet mass after MSC infusion.

Interestingly, an increasing number of studies have revealed that the underlying mechanism of MSC-induced islet restoration is closely related to the alteration of the intraislet macrophage phenotype. Macrophages are typically classified into two phenotypes: “classically activated” M1 macrophages and “alternatively activated” M2 macrophages, although the dichotomy is an oversimplification [16]. Generally, M1 macrophages secrete high levels of proinflammatory cytokines, such as tumor necrosis factor alpha (TNF*α*) and interleukin-1 beta (IL1*β*), and play crucial roles in the initiation and progression of inflammation. Ehses et al. found that there were more macrophages within islets from T2D patients and rodents than in healthy controls [17]. Further investigation demonstrated that islet-resident macrophages of normal animals exhibited an M2 phenotype, while accumulated macrophages within islets of T2D models polarized towards proinflammatory ones, leading to *β*-cell dysfunction [18]. In contrast, M2 macrophages are considered to be critical effector cells in the resolution of inflammation and the promotion of tissue repair [19, 20]. Emerging evidence has demonstrated a role of M2 macrophages during *β*-cell protection, repair, and regeneration [21–23]. Xiao et al. [24] found that M2 macrophages induced *β*-cell proliferation after pancreatic duct ligation and that depleting pancreatic macrophages with clodronate led to decreased *β*-cell replication. Notably, Yin et al. [25] found that in T2D mice, MSC-induced *β*-cell restoration resulted from alteration of the macrophage phenotype and the subsequent resolution of intraislet inflammation, indicating that unlike other antihyperglycemia agents, MSCs may protect *β*-cells from key aspects of the pathogenesis of T2D.

However, despite these encouraging results, the duration of efficacy of a single MSC infusion is relatively transient [9]. For example, Si et al. [9] found that the antidiabetic effect of a single MSC infusion was maintained for less than 4 weeks in T2D rats. Clinical trials also exhibited similar results [14, 26]. Liu et al. [14] found that the glycated hemoglobin levels of patients who had received MSC transplantation through one intravenous injection and one intrapancreatic endovascular injection decreased progressively in the first 3 months but gradually increased in the next 9 months.

Decitabine (DAC), a hypoethylating agent, is often used in the treatment of hematological disease. Recently, some studies have revealed the effectiveness of DAC in facilitating immune checkpoint therapy for solid tumors, indicating that DAC has immunoregulatory properties at low doses [27]. Additionally, growing information has indicated that low-dose DAC is able to prevent or treat some inflammatory illnesses, such as acute lung injury and atherosclerosis [28, 29]. Intriguingly, the preventive or therapeutic effect of DAC on these inflammatory diseases has also been ascribed to regulation of the macrophage phenotype. Cao et al. [29] found that low-dose DAC ameliorated atherosclerosis by suppressing proinflammatory macrophages. Thangavel et al. [28] showed that the combination treatment of DAC and another epigenetic modifier trichostatin A suppressed endotoxemia-induced acute lung injury by modulating the macrophage phenotype. Given the similar immunoregulatory capacities of DAC and MSCs, the combination of MSC infusion with DAC treatment may enhance the anti-inflammatory potency of MSCs, leading to an improved antidiabetic effect.

In this study, we investigated the therapeutic effect of a combinatory regimen of MSCs and DAC on streptozotocin (STZ)/high-fat diet-induced T2D mice. Mice were treated with a single intravenous infusion of human umbilical cord-derived MSCs (UC-MSCs) combined with five consecutive days of DAC administration, and they displayed relatively prolonged glucose homeostasis and a more durable restoration of *β*-cell mass than those given MSC infusion alone, which was accompanied by the polarization of the macrophage phenotype towards M2 in islets. In vitro, DAC enhanced the effect of UC-MSCs on macrophage polarization towards the M2 state. Taken together, our research demonstrated that the combinatory treatments of UC-MSCs and DAC may be a preferable therapeutic strategy for T2D.

## 2. Materials and Methods

### 2.1. Animal Experiments

Eight-week-old male C57BL/6J mice were purchased from the Chinese PLA General Hospital. Mice were kept in standard housing conditions (constant room temperature of 25°C, 12 h/12 h light-and-dark cycle, and free access to food and water). To induce the T2D model, mice were given high-fat diets (Sigma-Aldrich, St. Louis, MO, S0130) for 8 weeks and then were intraperitoneally injected with 90 mg/kg STZ (Sigma-Aldrich, St. Louis). Mice in the control group were fed normal chow diets (NCD) for 8 weeks. Mice whose random glucose levels were higher than 16.7 mmol/L for three consecutive days were considered to be T2D mice. Intraperitoneal glucose tolerance tests (IPGTTs) and intraperitoneal insulin tolerance tests (IPITTs) were also performed to verify the T2D model. For the IPGTT and IPITT, mice were fasted overnight and injected with glucose (1 g/kg) or insulin (1 U/kg), and then the blood glucose levels were detected at 30, 60, 90, and 120 min after the injection. T2D mice were randomly divided into 4 groups: (1) Mice in the DAC group were intraperitoneally administered 0.25 mg/kg DAC for 5 consecutive days. (2) Mice in the MSC group were infused with 1 × 10^6^ UC-MSCs suspended in 0.2 mL phosphate-buffered saline (PBS) via the tail vein. (3) Mice in the combined MSC and DAC group received both a single MSC infusion and 5-day DAC administration. (4) Mice in the T2D group were administered 0.2 mL PBS via the tail vein. Random blood glucose levels and weights were measured twice a week. IPGTT and IPITT were performed 1, 2, and 4 weeks after MSC infusion. The serum concentrations of insulin were detected by enzyme-linked immunosorbent assay (ELISA) kits (R&D Systems, Minneapolis, MN) according to the manufacturer's instructions. All experimental protocols were approved by the medical ethics committee of the Chinese PLA General Hospital.

### 2.2. Cell Culture

Human umbilical cords were obtained from women who gave birth in the Chinese PLA General Hospital with written informed consent. The procedure was approved by the Ethics Committee of the Chinese PLA General Hospital. UC-MSCs were isolated and characterized as described previously [30, 31]. Bone marrow-derived macrophages (BMDMs) isolated from the femur and tibia of 6-week-old C57BL/6J male mice were cultured in RMPI 1640 (Gibco, CA, USA) supplemented with 10% fetal calf serum (Gibco, CA, USA), 1% penicillin streptomycin (Gibco, USA), and 100 ng/mL M-CSF (R&D Systems, MN, USA) at 37°C in 5% CO_2_. After 5 days of culture, the identity of the cells was confirmed by anti-F4/80 flow cytometry. Peritoneal macrophages were collected from 6-week-old C57BL/6J male mice by peritoneal lavage with 1.5 mL RMPI 1640 and cultured with RMPI 1640 supplemented with 10% fetal calf serum and 1% penicillin streptomycin. The morphology of the macrophages was observed with phase-contrast microscopic images. BMDMs or peritoneal macrophages were seeded onto six-well plates, and then 100 ng/mL LPS (Sigma-Aldrich, USA) and 50 ng/mL IFN*γ* (R&D Systems, MN, USA) were added for 24 h to induce macrophages into the M1 phenotype. Then, macrophages were cultured with 4 × 10^4^ UC-MSCs in a Transwell system with or without adding DAC to the macrophage media.

### 2.3. Immunofluorescence Staining

The mouse pancreatic tissues were harvested 1, 2, or 4 weeks after MSC infusion. First, the mice anaesthetized with an intraperitoneal injection of 1% pentobarbital sodium (50 mg/kg) were perfused with PBS through the left ventricle, followed by 4% paraformaldehyde. Then, the pancreases were isolated, dehydrated with 30% sucrose/PB overnight, and embedded in optimal cutting temperature compound (OCT). Pancreatic sections (6 mm) were sliced by a microtome (Thermo Fisher Scientific) and incubated in a humidified chamber at 4°C overnight with primary antibodies against insulin (1/200, guinea pig, Sigma-Aldrich), glucagon (1/2,000, mouse, Abcam), Pdx1 (1/200, rabbit, CST), CD11c (1/200, mouse, Abcam), IL1*β* (1/100, rabbit, Abcam), F4/80 (1/200, rabbit, Sigma-Aldrich), and Fizz1 (1/200, rabbit, Abcam). After the sections were washed with PBS, they were incubated for 2 h with a secondary antibody (1 : 500; Alexa Fluor 488/594-conjugated secondary antibodies, Invitrogen) at room temperature. Nuclei were stained with DAPI (4′,6-diamidino-2-phenylindole, Sigma-Aldrich). The images were captured with a confocal laser scanning microscope (Olympus, Tokyo, Japan). Peritoneal macrophages spread on glass coverslips were fixed with 4% paraformaldehyde. The remaining steps were performed as described above.

### 2.4. CCK-8 Assay

BMDMs were seeded in a 96-well plate at 1 × 10^4^ cells/well and cultured with RMPI 1640 supplemented with 10% fetal calf serum, 1% penicillin streptomycin, and 100 ng/mL M-CSF for 24 h. Next, cells were treated with DAC at different concentrations (0, 1, 5, 10, 25, 50, 100, and 500 nmol/L) for 72 h. Then, the cells were incubated with fresh media containing CCK-8 for 30 min. The optical density was measured at OD450. The Cell Counting Kit-8 (CCK-8) Kit was purchased from DOJINDO Molecular Technologies.

### 2.5. Quantitative Real-Time Reverse Transcriptase Polymerase Chain Reaction

Total RNA was extracted from BMDMs using the TRIzol Reagent (Invitrogen) and quantified with the NanoDrop system (Thermo Fisher Scientific, Waltham, MA) according to the manufacturer's instructions. Then, RNA was reversely transcribed to cDNA with a reverse transcription kit (Thermo Fisher Scientific, Fremont, CA, http://www.thermo). Quantitative Real-Time Reverse Transcriptase Polymerase Chain Reaction (qRT-PCR) was performed in duplicate on a 7500 Real-Time PCR System with a SYBR Green PCR Master Mix (Applied Biosystems, Foster City, CA, http://www.appliedbiosystems.com). The thermal cycling program was 94°C for 3 min, followed by 40 cycles at 95°C for 15 s, 60°C for 15 s, and 72°C for 30 s. *β*-Actin was used as a reference gene. PCR primers are listed in Supplementary Table 1.

### 2.6. Western Blotting

BMDMs were washed twice with cold PBS and then lysed with Protein Extraction Reagent (CWBIO, China), which contained both protease inhibitor (CWBIO, China) and phosphatase inhibitors (Roche, Switzerland). The protein concentration in lysates was determined by the BCA assay (Thermo Fisher Scientific). Equal amounts of protein of each group were loaded onto SDS-PAGE gels and subsequently transferred to PVDF membranes. Next, membranes were blocked in 10% BSA and incubated with a primary antibody against Arg-1 (1 : 1000, Abcam, USA), iNOS (1 : 1000, Abcam, USA), PI3K (1 : 1000, CST, USA), p-AKT (1 : 1000, CST, USA), AKT (1 : 1000, CST, USA), *β*-tubulin (1 : 2000, ZSJQ-BIO, China), and GAPDH (1 : 2000, ZSJQ-BIO, China) overnight at 4°C. Then, the membranes were washed with TBST three times and incubated with the corresponding HRP-conjugated secondary antibodies for 1 h at room temperature. The blotting was detected by the ECL detection system (PPLYGEN, China) and analyzed by ImageJ software (NIH, MD). *β*-Tubulin and GAPDH were used as internal controls.

### 2.7. Flow Cytometry Analysis

BMDMs were harvested and suspended in PBS and subsequently incubated with a PE-conjugated anti-F4/80 antibody (eBioscience, USA) and isotype antibodies for 15 min at room temperature. The cells were washed two times with PBS, resuspended in 200 *μ*L PBS, and then analyzed by flow cytometry.

### 2.8. Statistical Analysis

All results were presented as the mean ± SD from at least 3 independent experiments. The results were analyzed using SPSS statistical software version 19 (SPSS Inc., IBM). Differences in the means were evaluated using the unpaired *t*-test or one-way ANOVA when required. *P* < 0.05 was considered statistically significant.

## 3. Results

### 3.1. UC-MSC Infusion Combined with DAC Displayed a More Prolonged Antidiabetic Effect Compared to UC-MSC Infusion Alone

**Figure 1:**
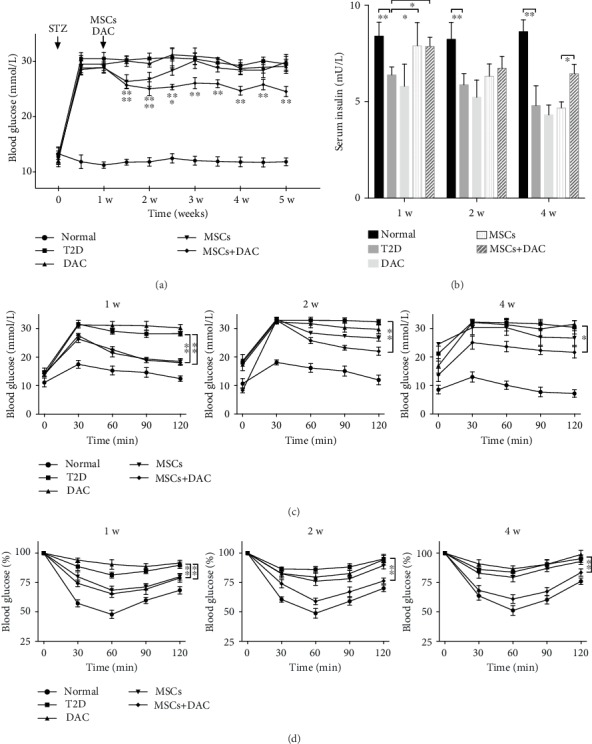
Human umbilical cord-derived-mesenchymal stem cell (UC-MSC) infusion combined with decitabine (DAC) exerted more prolonged antidiabetic effects than either treatment alone. (a) Blood glucose levels were measured consecutively after MSC infusion and DAC treatment. (b) Fasting serum insulin levels of five groups were detected by ELISA 1, 2, and 4 weeks after UC-MSC infusion and DAC treatment. (c and d) Glucose tolerance was assessed by the intraperitoneal glucose tolerance test (IPGTT) (c), and insulin tolerance was evaluated by the intraperitoneal insulin tolerance test (IPITT) 1, 2, and 4 weeks after UC-MSC infusion and DAC treatment. For the IPITT, the results were presented relative to the initial blood glucose concentration. Values of (a)–(d) are the mean ± SD; *n* = 6 mice per group; ^∗^*p* < 0.05 and ^∗∗^*p* < 0.01. Abbreviations: UC-MSC—human umbilical cord-derived mesenchymal stem cells; DAC—decitabine; STZ—streptozotocin; T2D—type 2 diabetes.

We investigated the antidiabetic effect of UC-MSCs and DAC in T2D mice induced by high-fat diets and STZ injection. First, we evaluated the T2D mouse model by measuring weight, blood glucose level, IPGTT, and IPITT. Before STZ injection, HFD-fed mice outweighed normal mice by 13.1 g on average. One week after STZ injection, blood glucose levels of the STZ-treated group were two times higher than those of normal mice (Supplementary Figure 1A). In addition, the IPGTT and IPITT further confirmed the success of the T2D model (Supplementary Figures 1B and 1C). Then, the T2D mice were divided into 4 groups and received different treatments. Mice in the DM group, the MSC group, and the DAC group received PBS infusion, UC-MSC infusion, and DAC treatment, respectively. Mice in the MSC plus DAC group (MD group) received both a single UC-MSC infusion and 5-day DAC injection. The chow diet-fed mice were the normal group. The T2D group (30.6 ± 0.9 mmol/L) showed persistent hyperglycemia and a gradual decrease in body weight, while blood glucose levels of the MSC group (26.8 ± 1.3 mmol/L) and MD group (25.1 ± 1.3 mmol/L) declined to a similar degree one week after MSC infusion. Nonetheless, DAC alone did not show a hypoglycemic effect (29.7 ± 0.7 mmol/L). As suggested by previous reports, the glucose level of the MSC group gradually increased and displayed no significant difference from that of the T2D group at the end of the study period (29.9 ± 1.4 mmol/L). Interestingly, the glucose level of the MD group was maintained at a lower level than that of the T2D group 4 weeks after treatment (24.6 ± 0.9 mmol/L) (Figure 1(a)). Accordingly, we detected serum insulin concentrations and found a continuous decrease in the fasting serum insulin levels in the T2D group, indicating progressive *β* cell dysfunction. MSC infusion transiently reversed the decline in serum insulin concentration, while the combination of MSCs and DAC exerted a prolonged enhancement (Figure 1(b)). In addition, the results of the IPGTT and IPITT indicated that the MSC group and the MD group showed a similar improvement in blood glucose metabolism and insulin sensitivity one week after treatment, but the MD group exhibited a better performance 4 weeks after treatment, consistent with the blood glucose levels (Figures 1(c) and 1(d)). Together, these results suggested that DAC prolongs the antidiabetic effect of MSCs to 4 weeks after treatment.

### 3.2. UC-MSC Infusion plus DAC Treatment Promoted a More Enduring Restoration of Islets Compared with UC-MSC Infusion Alone

**Figure 2:**
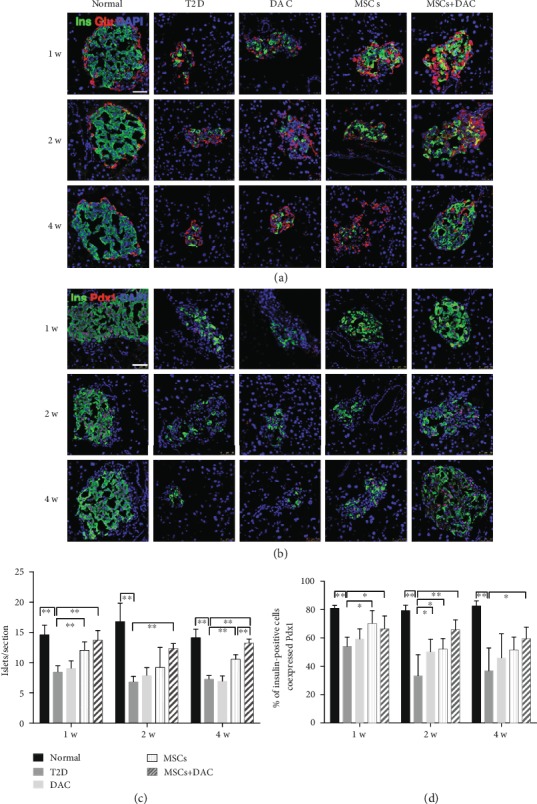
Human umbilical cord-derived- (UC-) mesenchymal stem cells (MSCs) combined with decitabine (DAC) exhibited an enduring restoration of pancreatic islet function. (a) Representative islets stained with antibodies against insulin (green) and glucagon (red) of five groups 1, 2, and 4 weeks after treatment. Nuclei were labeled with DAPI. Scale bars: 50 *μ*m. (b) Photomicrographs double stained with anti-insulin (green) and anti-Pdx1 (red) antibodies of the five groups 1, 2, and 4 weeks after treatment. Nuclei were labeled with DAPI. Scale bars: 50 *μ*m. (c) Quantification of islets observed per frozen section after immunofluorescence staining. (d) Quantification of insulin-positive cells coexpressing Pdx1. Quantification was determined by evaluating islets from at least 5 sections of each group. Data are shown as the mean ± SD, *n* = 5‐6 mice per group; ^∗^*p* < 0.05 and ^∗∗^*p* < 0.01.

To assess the effectiveness of MSCs and DAC in alleviating *β* cell dysfunction, we explored the dynamic changes of islets in each group via immunofluorescence staining. Mice of the T2D group showed a decreased mass of pancreatic islets, which were obviously abnormally structured. We also quantified the number of islets in each section of the pancreas. MSC infusion and UC-MSC+DAC treatment both ameliorated the morphological and structural damage to islets 1 week after treatment and increased the number of islets per section (Figure 2(a)). However, the restoration of islets in the MSC group was relatively transient. Four weeks after UC-MSC infusion, the islet mass was indistinguishable from that in the T2D group. Encouragingly, the MD group maintained a sustained restoration of islets 4 weeks after treatment (Figure 2(a)). The number of islets per section in the MD group (13.2 ± 0.7) 4 weeks after treatment was also significantly higher than that in the MSC group (10.5 ± 0.8) (Figure 2(c)). Next, we evaluated the expression of the vital *β* cell transcription factor Pdx1 by immunofluorescence. The ratio of insulin-positive cells expressing Pdx1 in the T2D group decreased to 55.4% one week after treatment and continuously declined over time compared with that of the normal group. UC-MSC infusion and UC-MSC+DAC treatments both elevated the ratio to approximately 70% one week after treatment. Four weeks after treatment, there were no significant differences in the ratio of insulin-positive cells expressing Pdx1 between the MSC group and T2D group, while the ratio in the MD group was still significantly higher than that in the T2D group (Figures 2(b) and 2(d)). These observations suggested that DAC effectively prolonged UC-MSC's effects on islet mass and function, which is in accordance with the observed blood glucose levels.

### 3.3. UC-MSC+DAC Treatment Induced M2 Macrophage Polarization and Mitigated Inflammation in Islets

**Figure 3:**
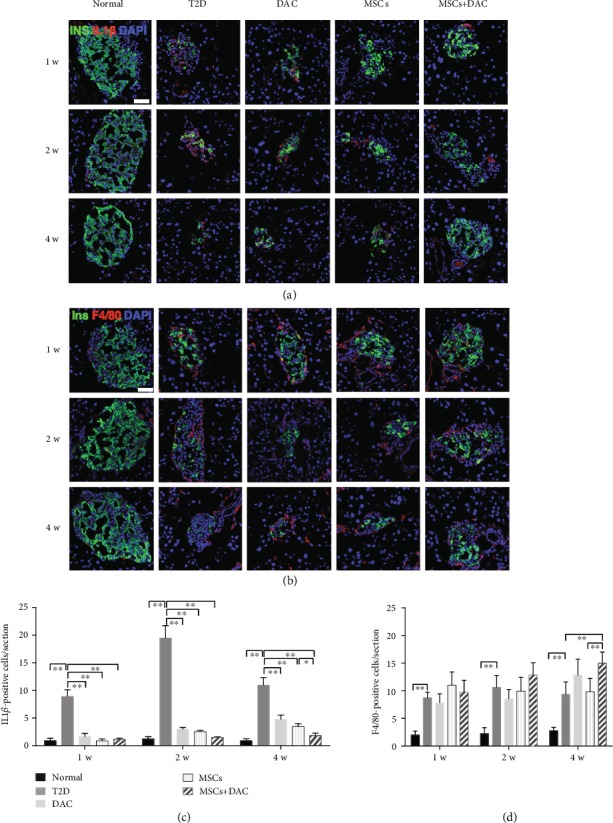
Human umbilical cord-derived- (UC-) mesenchymal stem cells (MSCs) combined with decitabine (DAC) mitigated intraislet IL1*β* expression and resulted in a subtle increase in the amount of macrophages. (a) Representative islets stained with antibodies against insulin (green) and IL1*β* (red) of five groups 1, 2, and 4 weeks after treatment. (b) Representative islets stained with antibodies against insulin (green) and F4/80 (red) in the five groups 1, 2, and 4 weeks after treatment. Nuclei were labeled with DAPI. Scale bars: 50 *μ*m. (c) Quantification of IL1*β*-positive cells per islet. (d) Quantification of F4/80-positive cells per islet. Quantification was determined by evaluating islets from at least 6 sections of each group. Data are shown as the mean ± SD, *n* = 6 mice per group; ^∗^*p* < 0.05 and ^∗∗^*p* < 0.01.

T2D is characterized by low-grade inflammation, and *β* cell dysfunction is strongly linked to the inflammatory microenvironment within islets [32]. IL1*β* is one of the most critical cytokines that account for progressive *β* cell dysfunction [32]. Therefore, we evaluated the expression of IL1*β* in islets. Similar to previous reports, there was an obvious accumulation of IL1*β* within diabetic islets. Compared with the normal group, the T2D group exhibited nearly 10-times higher levels of intraislet IL1*β*-positive cells. MSC infusion and MD treatment both remarkably reduced the intraislet expression of IL1*β* to a normal degree 1 week after treatment. As time went on, the intraislet expression of IL1*β* in the MSC group mildly increased. Four weeks after treatment, islets of the MD group showed the lowest expression of IL1*β* among the five groups (Figures 3(a) and 3(c)).

**Figure 4:**
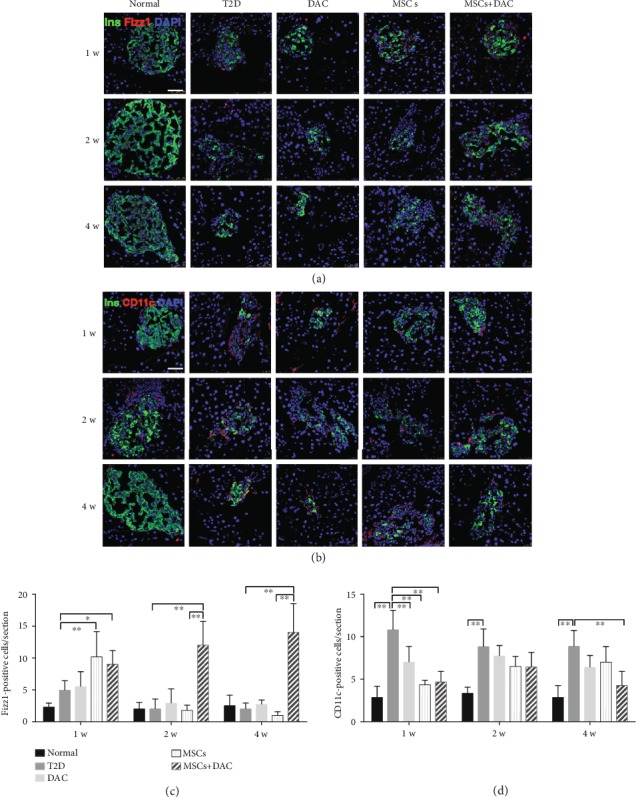
Human umbilical cord-derived- (UC-) mesenchymal stem cells (MSCs) combined with decitabine (DAC) induced M2 macrophage polarization in islets. (a) Representative islets stained with antibodies against insulin (green) and Fizz1 (red) of five groups 1, 2, and 4 weeks after treatment. Nuclei were labeled with DAPI. Scale bars: 50 *μ*m. (b) Representative islets stained with antibodies against insulin (green) and CD11c (red) of the five groups 1, 2, and 4 weeks after treatment. Nuclei were labeled with DAPI. Scale bars: 50 *μ*m. (c) Quantification of Fizz1-positive cells per islet. (d) Quantification of CD11c-positive cells per islet. Quantification was determined by evaluating islets from at least 6 sections of each group. Data are shown as the mean ± SD, *n* = 6 mice per group; ^∗^*p* < 0.05 and ^∗∗^*p* < 0.01.

Proinflammatory macrophages are considered to be the most important contributors of intraislet inflammatory cytokines such as IL1*β* and play essential roles in *β* cell dysfunction [22]; therefore, we investigated the number and phenotype of macrophages in islets. We found that compared with the number of F4/80-positive cells in the normal group, the number of F4/80-positive cells in islets improved in the T2D group, DAC group, MSC group, and MD group, but there were no obvious differences among the four groups (Figures 3(b) and 3(d)). Given that M1 macrophages and M2 macrophages play different roles in the progression and resolution of inflammation, the phenotype of intraislet macrophages was detected by immunofluorescence. Interestingly, there were prominent changes in the macrophage phenotypes after MSC infusion and DAC treatment. We counted the number of CD11c-positive cells and Fizz1-positive cells in each islet section. One week after treatments, there were many CD11c-positive cells and a few Fizz1-positive cells in the T2D group. The number of CD11c-positive cells in the MSC group and in the MD group remarkably decreased, and the number of Fizz1-positive cells increased compared with that in the T2D group (Figures 4(a) and 4(b)), indicating that M1 macrophages polarized towards M2 in the MSC and MD groups. Then, in the MSC group, the number of Fizz1-positive cells decreased over time. Four weeks after treatment, confocal micrographs showed obviously more Fizz1-positive macrophages and slightly fewer CD11c-positive macrophages in the MD group than in the MSC group (Figures 4(a)–4(d)). Collectively, these results indicated that the combination therapy of UC-MSCs plus DAC exerted a sustained effect on the polarization of intraislet macrophages towards M2, which probably contributed to the decreased accumulation of IL1*β* in islets.

### 3.4. UC-MSCs+DAC Further Polarized M1 Macrophages towards M2 Compared with UC-MSC Treatment Alone In Vitro

**Figure 5:**
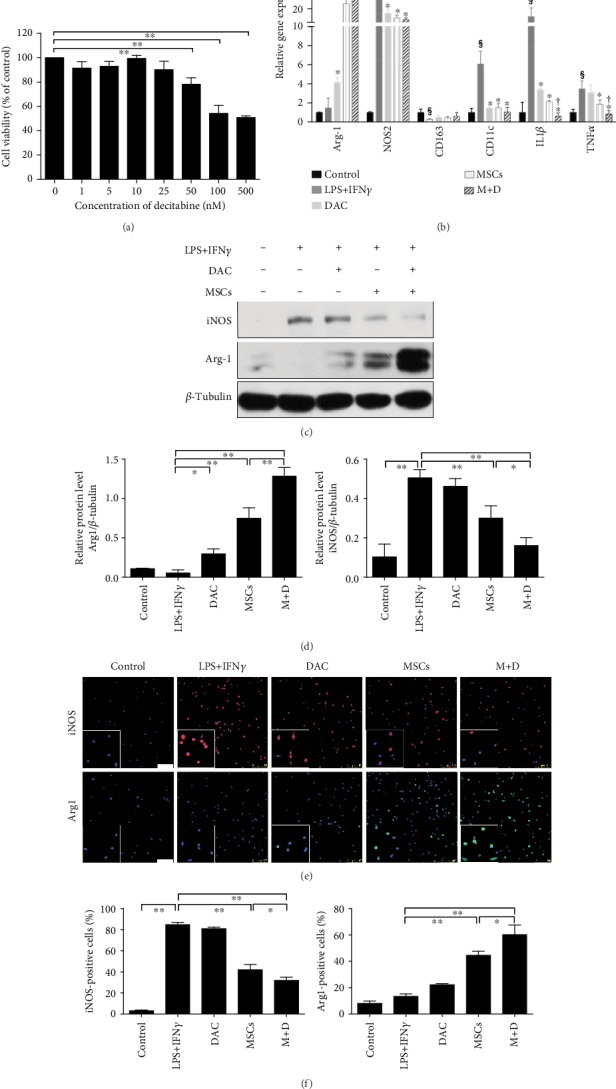
Human umbilical cord-derived- (UC-) mesenchymal stem cells (MSCs) combined with decitabine (DAC) further polarized M1 macrophages towards M2 compared with UC-MSCs alone in vitro. (a) BMDMs were incubated with DAC at concentrations ranging from 0 to 500 nmol/L for 72 h. Cell viability was determined by CCK-8 assay. The data are expressed as percentages of untreated control cells. (b–f) Macrophages were cultured alone (control) or in combination with LPS+IFN*γ* for 24 h, and then stimulated macrophages were cultured alone (LPS+IFN*γ*) or with UC-MSCs (MSCs), DAC (DAC), or both UC-MSCs and DAC (M+D) in a Transwell system for 72 h. (b) Quantitative reverse transcriptase polymerase chain reaction (RT-PCR) analysis of five groups. The results are presented relative to those of the control group, set as 1. (c and d) Immunoblotting analysis and quantification of Arg-1 and iNOS expression in BMDMs. *β*-Tubulin was used as a protein loading control. (e and f) Immunofluorescence and quantification of Arg-1 and iNOS expression in peritoneal macrophages. Nuclei were labeled with DAPI. Scale bars: 100 *μ*m. Values are the mean ± SD of three individual experiments. (a) and (c–f): ^∗^*p* < 0.05 and ^∗∗^*p* < 0.01. (b) ^§^*p* < 0.05 (vs. control); ^∗^*p* < 0.05 (vs. LPS+IFN*γ*); ^†^*p* < 0.05 (vs. MSC).

MSCs are well established to be capable of skewing macrophages towards the anti-inflammatory phenotype both in vivo and in vitro [5, 8, 33–36]. Given the alteration of the macrophage phenotype in vivo, we investigated whether DAC could augment the effect of MSCs on macrophage polarization in vitro. We used both mouse bone marrow-derived macrophages (BMDMs) and peritoneal macrophages, whose identities were evaluated by flow cytometry and immunofluorescence, respectively (Supplementary Figures 2A and 2B). First, to identify a proper dose of DAC in macrophages, we performed the CCK-8 assay and found that when macrophages were treated with DAC at concentrations of up to 25 nM for 72 h, the cell viability was not attenuated and maximized at 10 nM (Figure 5(a)). Additionally, we found that DAC could skew BMDMs into M2 as previous researches showed [28, 29, 37], and DAC at the dose of 10 nmol/L achieved the optimal effect on macrophage polarization in our in vitro model (data not shown). Therefore, 10 nM DAC was applied in the in vitro study. We utilized LPS (100 ng/mL) and IFN*γ* (50 ng/mL) to induce macrophages towards M1, and then M1 macrophages were cultured with UC-MSCs in a Transwell system in the presence or absence of DAC in macrophage media. The morphology of BMDMs changed after LPS+IFN*γ* treatment (Supplementary Figure 2C). RT-PCR analysis showed increased expression of Arg-1, an M2 macrophage-related gene, and decreased expression of M1 macrophage-related genes (NOS2, TNF*α*, and IL1*β*) in macrophages treated with MSCs+DAC compared to cells treated with MSCs or DAC alone (Figure 5(b)). Similarly, western blot analysis showed an upregulation of Arg-1 expression in both the DAC group and the MSC group compared with that in the LPS+IFN*γ* group, and MD treatment led to a significant augmentation of Arg-1 expression relative to treatment with MSCs or DAC alone. Additionally, MD treatment further downregulated the expression of iNOS (Figures 5(c) and 5(d)). In parallel, the results were confirmed by immunofluorescence analysis of peritoneal macrophages (Figures 5(e) and 5(f)). In conclusion, these results revealed that the combination of UC-MSCs and DAC could further polarize M1 macrophages towards M2 macrophages.

### 3.5. UC-MSCs+DAC Regulated Macrophage Polarization via the PI3K/AKT Signaling Pathway

**Figure 6:**
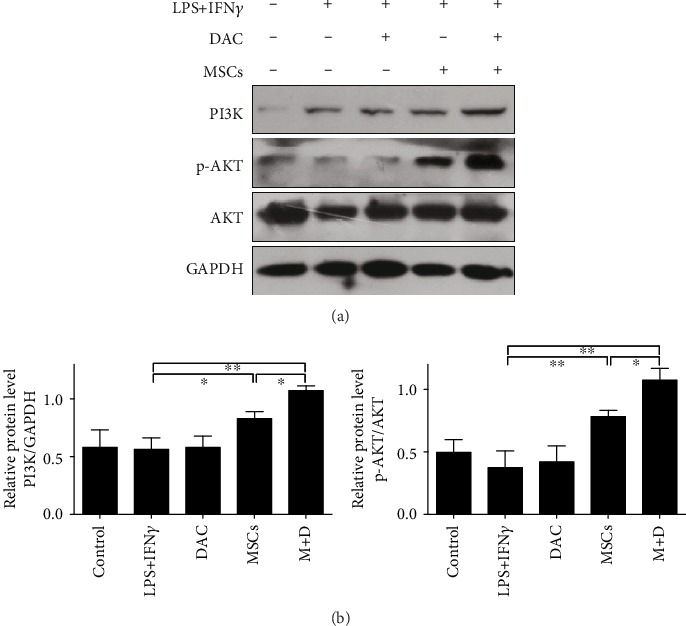
UC-MSCs combined with decitabine induced activation of the PI3K/AKT pathway. (a and b) Immunoblotting analysis and quantification of phosphorylated AKT (p-AKT), total AKT, PI3K, and GAPDH expression in BMDMs. GAPDH was used as a protein loading control. Values are the mean ± SD of three individual experiments. ^∗^*p* < 0.05 and ^∗∗^*p* < 0.01.

**Figure 7:**
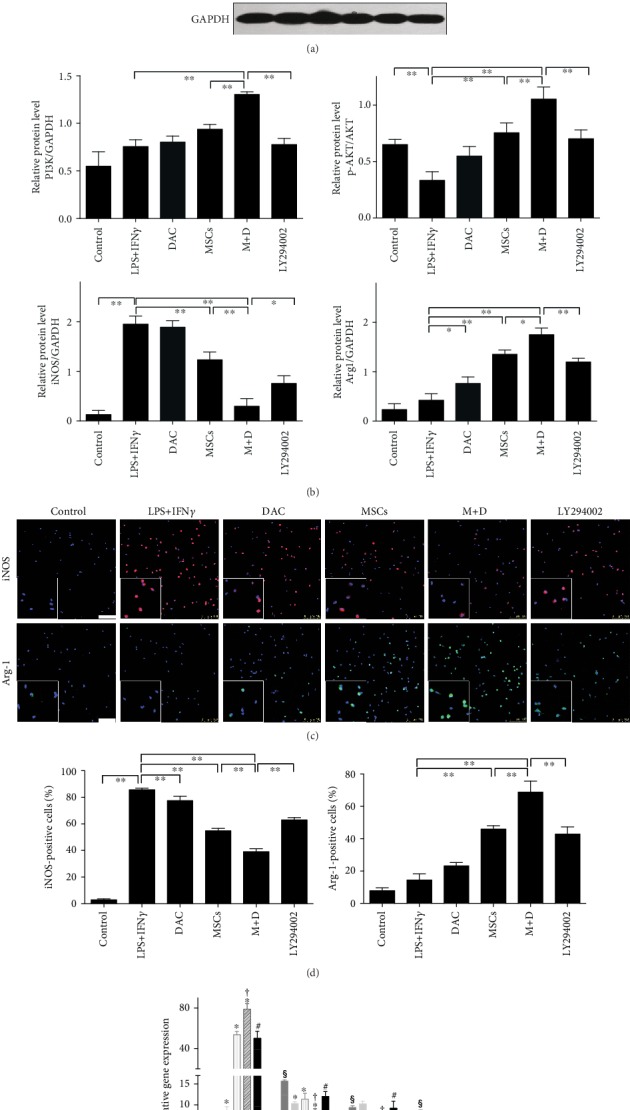
Human umbilical cord-derived- (UC-) mesenchymal stem cells (MSCs) combined with decitabine (DAC) regulated macrophage polarization via the PI3K/AKT signaling pathway. (a and b) Immunoblotting analysis and quantification of PI3K, phosphorylated AKT (p-AKT), total AKT, iNOS, Arg-1, and GAPDH in BMDMs. GAPDH was used as a protein loading control. (c and d) Immunofluorescence and quantification of Arg-1 and iNOS in peritoneal macrophages. Nuclei were labeled with DAPI. Scale bars: 100 *μ*m. (e) RT-PCR analysis of Arg-1, NOS2, IL1*β*, and TNF*α* expression in the five groups. The results are presented relative to those of the control group, set as 1. Values are the mean ± SD of three individual experiments. (a–d) ^∗^*p* < 0.05 and ^∗∗^*p* < 0.01. (e) ^§^*p* < 0.05 (vs. control); ^∗^*p* < 0.05 (vs. LPS+IFN*γ*); ^†^*p* < 0.05 (vs. MSC); ^#^*p* < 0.05 (vs. M+D).

To confirm the underlying mechanism of macrophage polarization by UC-MSC+DAC treatment, we investigated the possible signaling pathway. Studies have found that the PI3K/AKT signaling pathway is essential in macrophage activation and polarization [38, 39]. The activation of the PI3K/AKT pathway leads to less inducible NO synthase and decreased proinflammatory cytokine expression [40, 41], while the downregulation of the PI3K/AKT pathway results in a reduced expression of a series of M2 genes, including Arg-1 [42]. We therefore detected the expression of molecules in the PI3K/AKT pathway by western blot. After MD treatment, macrophages showed an obvious increase in PI3K and p-AKT expression (Figures 6(a) and 6(b)). Next, we used LY294002, an inhibitor of PI3K, to block the PI3K/AKT pathway in BMDMs and peritoneal macrophages. Western blot and immunofluorescence analysis both showed that LY294002 partly suppressed Arg-1 expression and elevated iNOS expression (Figures 7(a)–7(d)). Due to the inability to suppress the PI3K/AKT pathway completely, Arg-1 expression remained in the Ly294002 group. In addition, RT-PCR validated the results and revealed an upregulation of inflammatory cytokines (TNF*α* and IL1*β*) after Ly294002 treatment (Figure 7(e)). These results suggested that UC-MSCs+DAC polarized macrophages to the anti-inflammatory type at least partially through the PI3K/AKT pathway.

**Figure 8:**
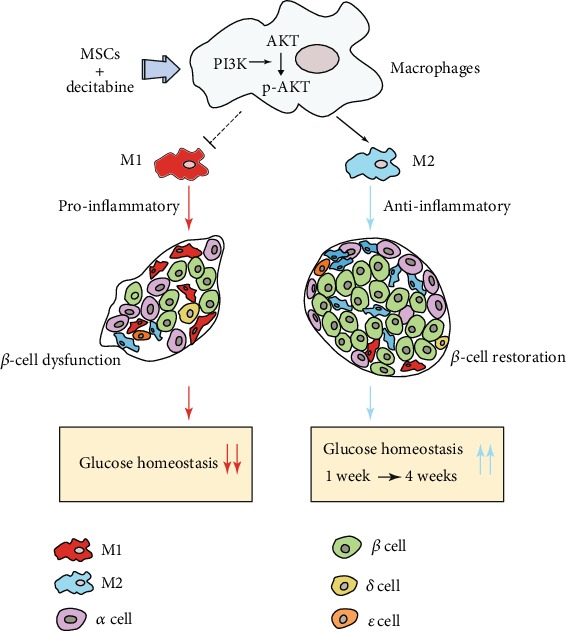
Schematic diagram of the effect of human umbilical cord-derived- (UC-) mesenchymal stem cells (MSCs) combined with decitabine (DAC) on T2D mice. The accumulation of proinflammatory M1 macrophages resulted in *β*-cell dysfunction and impaired glucose homeostasis in T2D. UC-MSCs combined with DAC induced sustained macrophage polarization towards M2 via the PI3K/AKT signaling pathway, leading to prolonged *β*-cell restoration and improvement of glucose homeostasis.

All together, these results, both in vivo and in vitro, demonstrated that UC-MSCs combined with DAC polarized macrophages towards the anti-inflammatory type, leading to prolonged *β*-cell restoration and the improvement of glucose homeostasis in T2D mice (Figure 8).

## 4. Discussion

T2D has become a major public health concern worldwide. Due to the regenerative properties and potent immunoregulatory capabilities, MSCs are considered ideal candidate cells for T2D treatment [43], but the efficacy of a single MSC infusion has been relatively transient in both animal experiments [9] and clinical trials [14, 26]. Therefore, the search for strategies to enhance or prolong the efficacy of MSCs is meaningful. To the best of our knowledge, this is the first study that demonstrated the combination of UC-MSCs and DAC displayed a prolonged antidiabetic effect by promoting sustained restoration of islets compared with UC-MSC infusion alone, thereby enriching the MSC treatment.

MSCs, which are considered to possess the ability to treat T2D fundamentally by both ameliorating insulin resistance and restoring islet function [9, 11, 43], have promising application prospects. However, the limited hypoglycemic effect after a single MSC infusion will limit its clinical use. At present, many researchers are devoted to enhancing or prolonging the therapeutic effect of MSCs via various methods in the treatment of T2D. Hu et al. [44] reported that the combination of two MSC infusions with 10 weeks of intragastric administration of sitagliptin showed improved glycemic homeostasis in T2D rats. Compared with the combination of MSC infusion with everyday oral medicine treatment, combinatory therapy of MSCs and DAC may simplify MSC application in clinical use. Hao et al. [10] found that multiple intravenous infusions of bone marrow MSCs at 2-week intervals sustainably reversed hyperglycemia in T2D rats. However, multiple infusions of MSCs may increase the risk of potential side effects, such as pulmonary and upper respiratory adverse events, allergic events, and tumor formation. Our findings may help decrease the frequency of MSC infusion in future clinical applications and therefore reduce the potential risks of MSC infusions. Other studies have combined MSC infusion with hyperbaric oxygen [45], adjusting the approaches of MSC infusion [10, 46], but no study has prolonged the therapeutic duration of a single MSC infusion to 4 weeks in vivo.

Macrophages are pivotal effectors in the onset and progression of the islet pathological process of T2D [21, 22, 47]. Generally, macrophages are classified into two phenotypes: “classically activated” M1 macrophages and “alternatively activated” M2 macrophages. In response to chemokines produced by *β*-cells, M1-like monocytes/macrophages infiltrated into islets and secreted large amounts of proinflammatory cytokines, leading to *β* cell dysfunction and loss [18, 47]. In contrast, emerging data have demonstrated that M2 macrophages in islets contribute to *β*-cell protection, repair, and regeneration after acute or chronic islet injury [23, 24, 48, 49]. Criscimanna et al. [49] found that proper macrophage polarization towards M2 was essential for pancreatic regeneration. Emerging data has shown that MSC-induced macrophage polarization plays an important role in the protection of islet function in type 2 diabetic mice [50]. Yin et al. [25] found that UC-MSCs secreted IL6 to polarize macrophages towards M2. In vivo UC-MSC infusion led to more M2 macrophages in pancreatic islets and therefore alleviated islet dysfunction in T2D mice, and inhibition of IL6 production in MSCs resulted in fewer intraislet M2 macrophages and attenuated islet restoration, indicating that M2 macrophages play an important role in MSC-induced islet restoration. In accordance with previous research, we observed a substantial elevation in M1 macrophages in islets of T2D mice. However, 1 week after UC-MSC infusion, M1 macrophages decreased, while M2 macrophages dramatically increased within islets, and the alteration became insignificant 4 weeks after the single MSC infusion. Interestingly, unlike UC-MSC infusion alone, DAC plus MSCs further prolonged MSC-induced intraislet macrophage polarization. Notably, the polarization of intraislet macrophages towards M2 in vivo was in parallel with the restoration of islets in the MD group, and the in vitro study also showed that MSCs plus DAC further polarized macrophages towards the M2 phenotype. Hence, although we did not deplete macrophages to observe islet changes after MSC plus DAC treatment, the above results suggested that MD-induced islet restoration was probably ascribed to intraislet macrophage polarization.

DAC, a kind of epigenetic modifier, is able to induce DNA hypomethylation and reactivate gene expression. Although DAC is cytotoxic at high doses, at low doses, it has been proven to be safe and is approved by the U.S. Food and Drug Administration to treat myelodysplastic syndrome and chronic myelomonocytic leukemia. In this study, DAC (0.25 mg/kg) was administered by intraperitoneal injection for 5 consecutive days. McCabe et al. [51] showed that mice treated with 0.25 mg/kg DAC for 18 weeks did not suffer from any visible side effects, indicating that DAC at this low dose was safe. In parallel, we observed no significant differences in body weight and food intake between the T2D group and the DAC group (data not shown). Despite hematological diseases, increasing data demonstrate that DAC is a promising therapeutic modality for other diseases. Recent studies found that DAC was effective in the treatment of solid tumors by promoting immune checkpoint therapy and sensitizing chemotherapeutic drugs [52]. DAC also possesses immunoregulatory effects on immune cells, including macrophages and Treg cells at low doses [28, 29, 53], hence exerting a therapeutic effect on autoimmune or inflammatory illness. Some studies have combined DAC with drugs that possess a similar immunomodulatory effect as DAC to treat inflammatory diseases. Thangavel et al. [28] reported that DAC or a histone deacetylase inhibitor Trichostatin A (TSA) alone only had a limited effect on macrophage polarization, but the combination treatment of DAC and TSA led to substantial macrophage phenotypic changes and alleviated endotoxemia-induced acute lung injury in C57/BL mice. Our research also found that DAC showed a temperate and mild influence on macrophage polarization, but the combination of DAC with UC-MSCs exhibited dramatic phenotypic changes in macrophages in vitro, demonstrating a promising method to potentiate MSC therapeutic efficacy as well as an expanding application of DAC.

In view of the alteration of the macrophage phenotype within islets, we investigated the effect of UC-MSCs and DAC on macrophage polarization in vitro. We found that the combination of DAC with UC-MSCs resulted in dramatic phenotypic changes in macrophages. Further investigation found that MSC+DAC-induced macrophage polarization was partly induced by the PI3K/AKT pathway, which is critical in the control of macrophage polarization [38, 39, 41, 54]. Li et al. [55] reported that DAC could lead to the hypomethylation of the VSIG4 gene and therefore enhance the PI3K/AKT-STAT3 signaling pathway, leading to the inhibition of M1 macrophage activation. Given that DAC is a demethylation agent, in future studies, we should determine whether the demethylation effect of DAC directly affects the expression of PI3K/AKT and thus causes the polarization of macrophages.

## 5. Conclusions

In summary, our study for the first time revealed that a single UC-MSC infusion in combination with low-dose DAC effectively prolonged the therapeutic effect of UC-MSCs in T2D mice via the sustained restoration of islets, which was correlated with elevated intraislet M2 macrophages and decreased M1 macrophages. In vitro, we found that UC-MSCs combined with DAC was more efficacious in promoting M1 macrophage polarization towards M2 by augmenting the PI3K/AKT pathway. These results demonstrated that the combination of UC-MSCs and DAC was a promising strategy for T2D treatments.

## Data Availability

The datasets used and/or analyzed during the study are available from the first author upon request.

## Supplementary Materials

Supplementary Materials Supplementary Figure 1: identification of T2D mice models. Supplementary Figure 2: identification of bone marrow-derived macrophages (BMDMs) and peritoneal macrophages. Supplementary Table 1: primer sequences of target genes (mouse).
