# Supplementary material for: Low-Dose Decitabine Assists Human Umbilical Cord-Derived Mesenchymal Stem Cells in Protecting β Cells via the Modulation of the Macrophage Phenotype in Type 2 Diabetic Mice
Source: Stem Cells Int. 2020 Apr 3;2020:4689798. doi: 10.1155/2020/4689798 (PMC7157805; doi:10.1155/2020/4689798)
Supplement: Supplementary Materials — Supplementary Figure 1: identification of T2D mice models. Supplementary Figure 2: identification of bone marrow-derived macrophages (BMDMs) and peritoneal macrophages. Supplementary Table 1: primer sequences of target genes (mouse). [file 4689798.f1.zip › mat.4689798.v2.pdf]

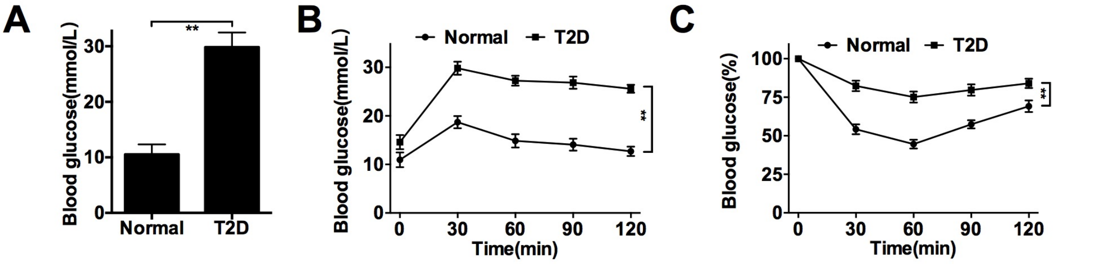

Supplementary Figure 1. Identification of T2D mice models.

The T2D mice model was induced by 8-week HFD and a single introperitoneal injection of STZ. (A) The random blood glucose levels of normal mice and T2D mice after one week of STZ injection. (B, C) One week after STZ injection, the glucose tolerance of normal mice and T2D mice was assessed by the intraperitoneal glucose tolerance test [IPGTT] (B), and insulin tolerance was evaluated by the intraperitoneal insulin tolerance test [IPITT] (C). Data are shown as the mean±SD, n=6 mice per group; *P<0.05 and **P<0.01.

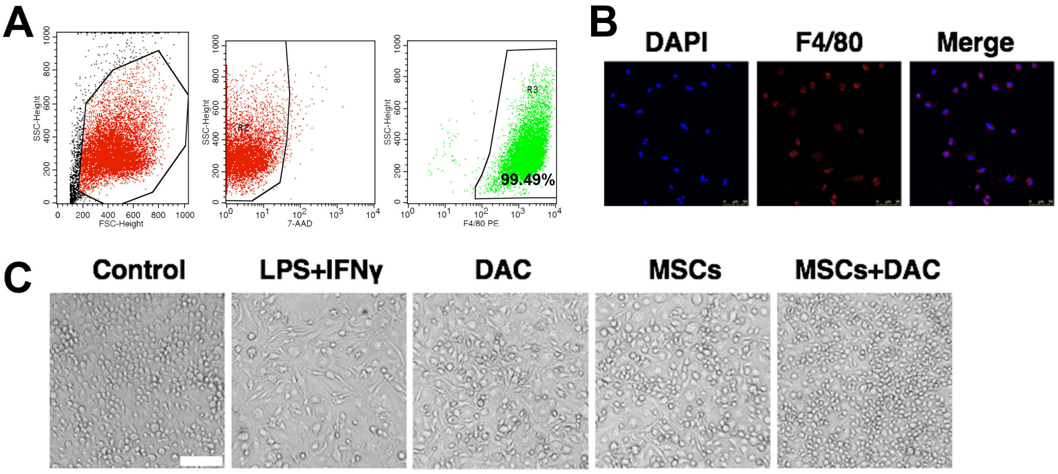

Supplementary Figure2. Identification of bone marrow-derived macrophages (BMDMs) and peritoneal macrophages.

(A) Identification of BMDMs by flow cytometry. BMDMs were stained with F4/80-PE and then were analyzed by flow cytometry. (B) Identification of peritoneal macrophages by immunofluorescence. Peritoneal macrophages were stained with F4/80. (C) Macrophages were cultured alone(Control) or in stimulation with LPS + IFN γ for 24h, and then stimulated macrophages were cultured alone(LPS+IFN γ) or either with UC-MSCs in a trans-well system(MSC) or DAC(DAC), or by treatment with UC-MSCs and DAC(M+D) together for 72h. (C) The morphological changes of BMDMs of fives groups. Images of A-C are representatives of three independent experiments.

Supplementary Table 1.Primer sequences of target genes (mice)

Genes	Primer sequence (5’-3’)
Arg1	For: CCAGCA TTCACCCCGGCGAC<br>Rev: GCCCTTGGGAGGAGAAGGCGT

NOS2	For: TCACCTTCGAGGGCAGCCGA<br>Rev: TCCGTGGCAAAGCGAGCCAG
CD163	For:GGGTCATTCAGAGGCACACTG<br>Rev:GCTGGCTGTCCTGTCAAGGCT
CD11c	For: ACGTCAGTACAAGGAGATGTTGGA<br>Rev: ATCCTATTGCAGAATGCTTCTTTACC
IL1β	For: TGGGCCTCAAAGGAAAGAAT<br>Rev: CAGGCTTGTGCTCTGCTTGT
TNFα	For: CCAGACCCTCACACTCAGATC<br>Rev: CACTTGGTGGTTTGCTACGAC
β-actin	For：AGTGTGACGTTGACA TCCGT<br>Rev：GCAGCTCAGTAACAGTCCGC
